# MicroRNA-155 is a potential molecular marker of natural killer/T-cell lymphoma

**DOI:** 10.18632/oncotarget.10780

**Published:** 2016-07-22

**Authors:** Xudong Zhang, Weiguo Ji, Ruixia Huang, Lifeng Li, Xinhua Wang, Ling Li, Xiaorui Fu, Zhenchang Sun, Zhaoming Li, Qingjiang Chen, Mingzhi Zhang

**Affiliations:** ^1^ Department of Oncology, The First Affiliated Hospital of Zhengzhou University, Zhengzhou 450008, China; ^2^ Department of Oncology, Third Affiliated Hospital of Henan College of Traditional Chinese Medicine, Zhengzhou 450008, China; ^3^ Department of Oncology, Institute of Clinical Medicine, The First Affiliated Hospital of Zhengzhou University, Zhengzhou 450008, China

**Keywords:** natural killer/T-cell lymphoma, Solexa high-throughput sequencing, miRNA, miRNA-155

## Abstract

Natural killer/T-cell lymphoma (NKTCL) is characterized by its highly aggressive nature and rapid progression. MicroRNAs (miRNAs) play key roles in the development of NKTCL. We utilized next-generation Solexa high-throughput sequencing to compare miRNA expression in the SNK-6 and YTS NKTCL cell lines with expression in normal NK cells. We found that 195 miRNAs were upregulated in the SNK-6 cells and 286 miRNAs were upregulated in the YTS cells. Based on those results, we selected six miRNAs, including miRNA-155, and confirmed their expression using real-time polymerase chain reaction. Expression of miRNA-155 was higher in SNK-6 and YKS cells than in normal NK cells. We next determined the levels of miRNA-155 in the serum of healthy individuals and NKTCL patients, and correlated its expression with clinical parameters and inflammatory factors detected using enzyme-linked immunoabsorbent assays. Levels of miRNA-155 were higher in NKTCL patients’ serum than in serum from healthy individuals. miRNA-155 expression was higher in patients with stable or progressive disease (SD+PD) than in those with partial or complete remission (PR+CR). While further studies are needed to clarify the underlying molecular mechanisms, it appears miRNA-155 may be a molecular marker of NKTCL.

## INTRODUCTION

Natural killer/T-cell lymphoma (NKTCL) is a subtype of non-Hodgkin's lymphoma, which has a high incidence in East Asia and Latin America but a low incidence in the United States and Europe [1]. NKTCL is a tumor with a very specific morphology, immunophenotype, and biological behavior. Among its different types, the nasal form accounts for 70%–90% of the incidence of extranodal NKTCL, the most common category of NKTCL [2]. This tumor is characterized by its highly aggressive nature, rapid progression, short survival time, and poor prognosis [3]. In addition, because of controversy over treatment methods, it has been a focus of intensive study among oncologists.

MicroRNAs (miRNAs) are a hot research topic in the field of lymphoma molecular biology. Among their many functions, miRNAs regulate the expression of genes involved in tumor growth and development [4]. Numerous trials have indicated that miRNAs may play key roles in the development of NKTCL. For example, miRNA-21 and miRNA-155 are highly expressed in NKTCL cell lines where they activate the AKT signaling pathway to downregulate tumor suppressor genes, leading to tumor cell proliferation [5]. Others have previously explored whether miRNA-221 levels in circulating blood can be used as a biomarker for the diagnosis and prognosis of NKTCL [6], and shown that miRNA-146a downregulates the activity of NF-KB by regulating the target molecule TRAF6, which is a tumor suppressor gene in NKTCL and is related to patient prognosis [7]. Ti et al. [8] first detected the abnormal expression profile of miRNAs in NKTCL. The unusual expression of some miRNAs was also found to be associated with signaling pathways that affect tumor growth and proliferation, such as MYC activation [9] and Epstein–Barr (EB) virus infection [10].

In this study, we applied next-generation Solexa high-throughput sequencing technology to examine expression of miRNAs in the NKTCL cell lines SNK-6 and YTS, as well as in normal NK cells, and carried out further sequencing to confirm differentially expressed genes. In contrast to our previous findings, we confirmed that miRNA-155 is a key molecule affecting the biological characteristics of NKTCL [11]. We also detected the level of miRNA-155 in serum from healthy individuals and NKTCL patients, and found that the expression of miRNA-155 in NKTCL patients was higher than in healthy controls. Combined with the data on clinical factors and immune indicators of these patients, we demonstrated that miRNA-155 plays an important role in the emergence and development of NKTCL, making it a potential clinical diagnostic marker and molecular target of NKTCL.

## RESULTS

### Clustering analysis of miRNA sequencing

Before sequencing, RNA samples were tested in an Agilent 2100 bioanalyzer for analysis of RNA concentrations, fragment sizes, RIN, and 28S/18S ratios. All RNA samples were of high purity and integrity and met the requirements for constructing sequencing databases. The three cell lines were each subjected to high-throughput deep sequencing; as a result, 7,476,202, 6,903,218, and 8,458,056 clean reads were produced, with the vast majority of the sequences being 18-24 nt in length. Difference comparison, cluster analysis, and analysis of additional biological information of the known miRNAs was used to compare SNK-6, YTS, and normal NK cells. When SNK cells were compared with normal NK cells, among the 388 known miRNAs, 195 had upregulated expression, 67 had downregulated expression, and 126 showed no difference. When YTS cells were compared with normal NK cells, among the 457 known miRNAs, 286 showed upregulated expression, 64 showed downregulated expression, and 102 showed no difference.

It would be difficult to identify and evaluate the biological functions for all of these differentially expressed miRNAs. Hence, the miRNAs with differential expression were screened to identify candidates with (1) a fold change (log2 NK/SNK-6 or YTS) of >1.0 or <−1.0, and p-value <0.01; and either (2) a confirmed or potential relationship to the biological characteristics of lymphoma cells, such as those involved in cellular processes, signaling pathways, drug metabolism, immune processes, and viral infection, among others; OR (3) a normalized standard value of miRNA >50, and differential fold change of > or < 5.0. When SNK-6 was compared with normal NK cells, 25 candidate miRNAs meeting these criteria were upregulated and 12 were downregulated. In addition, when YTS was compared with normal NK cells, there were 30 upregulated and 12 downregulated miRNAs (Supplementary Tables S2 and S3). Part of the differential expression clustering analysis of small RNAs in NK, SNK-6, and YTS cells is shown in Supplementary Figure S1.

### Identification of differentially expressed miRNAs

Six different miRNAs (miRNA-34a, miRNA-34b, miRNA-34c, miRNA-155, miRNA-21, miRNA-221), which were expressed at high, intermediate, and low levels in samples from the NKTCL cell lines SNK-6 and YKS, were selected for analyses of their expression using real-time PCR. Expression of all six of these miRNAs could be detected. The relative expression of miRNA-34a in the SNK-6 and YKS cells was −5.40 ± 0.42 and −5.10 ± 0.44, respectively, which was lower (p<0.05) than normal NK cells. The effective fragment of miRNA-34b in SNK-6 and YKS cells, as well as normal NK cells, was not expressed. The relative expression of miRNA-21 in the SNK-6 and YKS cells was 3.18 ± 0.26 and 4.08 ± 0.39, respectively, being higher (p<0.05) than in normal NK cells. In addition, the expression of miRNA-155 in SNK-6 and YKS cells was higher (p<0.05) than in normal NK cells; and this expression was also clearly higher (p<0.05) in SNK-6 cells than in YKS cells. These results were somewhat consistent with the small RNA sequencing results, with the exception of miRNA-34c, which had a relative expression in the SNK-6 and YKS cells of 0.17 ± 1.6 and 0.04 ± 0.81, respectively, and which did not differ (p>0.05) from normal NK cells. In addition, the sequencing results for miRNA-211 in the SNK-6 and YKS cells showed decreases in expression, whereas real-time PCR analysis showed contradictory results. The relative expression in SNK-6 cells was 2.21 ± 0.54, which was higher (p<0.05) than in normal NK cells, while expression in YSK cells was 1.04 ± 0.09, showing no difference (p>0.05; Table 1).

**Table 1:** qRT-PCR validation and expression analysis of miRNAs in SNK-6 and YTS cells

miRNA	SNK-6		YTS	
	−ΔΔCt	fold_change	−ΔΔCt	fold_change
miRNA-34a	−5.40 ±0.42^a^	−5.56	−5.10 ±0.44^a^	−4.85
miRNA-34b	Not detected	Not detected	Not detected	Not detected
miRNA-34c	0.17 ±1.69 b	0.82	0.04 ±0.81 b	2.41
miRNA-221	2.21 ±0.54 a	−1.48	1.04 ±0.09 b	−3.76
miRNA-21	3.18 ±0.26 a	2.97	4.08 ±0.39 a	1.05
miRNA-155	6.64 ±0.36 ac	2.62	2.61 ±0.45 a	2.12

A compared with normal NK cells, *p*<0.05;

b compared with normal NK cells, *p*>0.05;

c compared with YTS cells, *p*<0.05.

### Expression of serum miRNA-155 in patients with NKTCL

**Figure 1:**
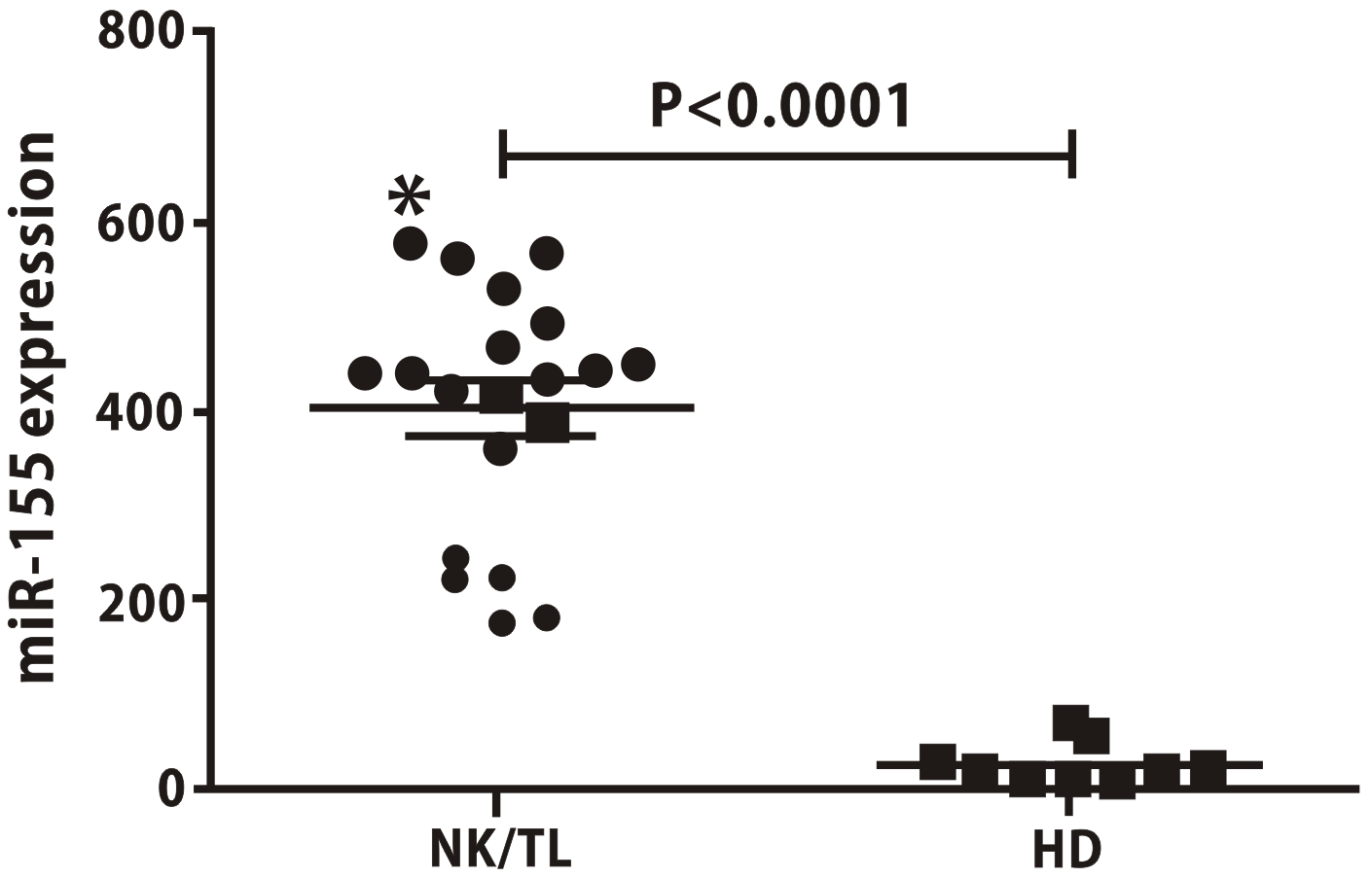
Serum levels of miR-155 are higher in NKTCL patients than in healthy donors The expression of miRNA-155 in NKTCL patients was higher than in healthy controls (402.13±128.30 vs.24.16±20.14, *p*<0.05).

Based on the analyses of 20 NK/T lymphoma patients and 10 healthy individuals, the expression of miRNA-155 in the patient group (402.13 ± 128.30) was higher than in the healthy group (24.16 ± 20.14), as shown in Figure 1.

### Serum miRNA-155 expression and clinical parameters

**Figure 2:**
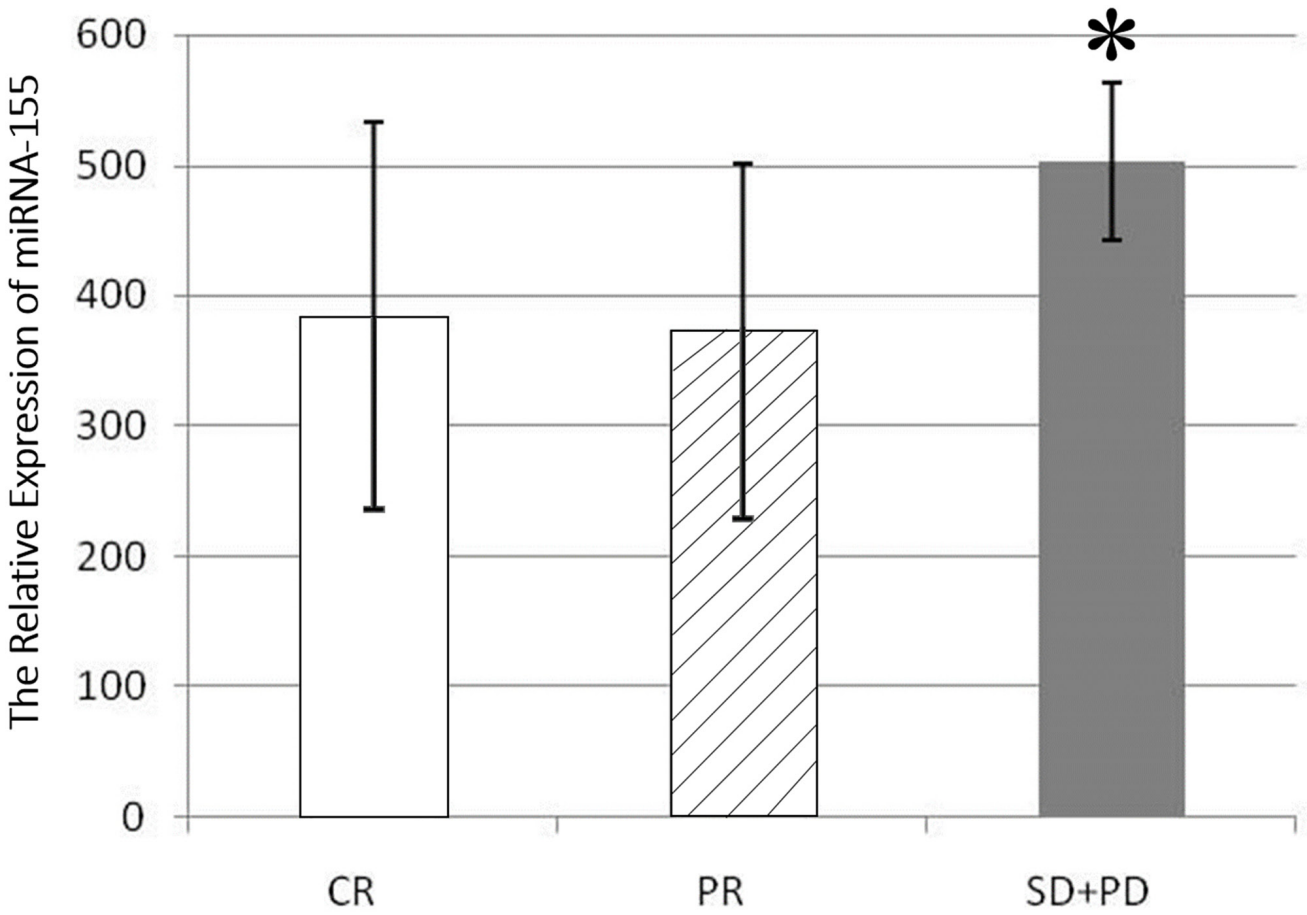
miR-155 expression in the serum of patients in different efficacy groups The expression in the SD+PD group was higher than in CR group (503.03±60.71 *vs.* 384.37±148.25, *p*<0.05), and PR group (503.03±60.71 *vs.* 373.51±127.4, *p*<0.05).

According to the evaluation of the therapeutic efficacy (two cycles), the lymphoma patients could be divided into a CR group, a PR group, and a group in which treatment was ineffective (SD+PD). Expression of miRNA-155 in the plasma of the SD+PD patients was higher than in the other two groups (P<0.05), while the comparison between the PR group and the CR group showed no difference (Figure 2). These results indicate that miRNA-155 is a potential molecular marker of therapeutic efficacy in NKTCL patients.

### Correlation of inflammatory cytokines and miR-155 in NKTCL

**Figure 3:**
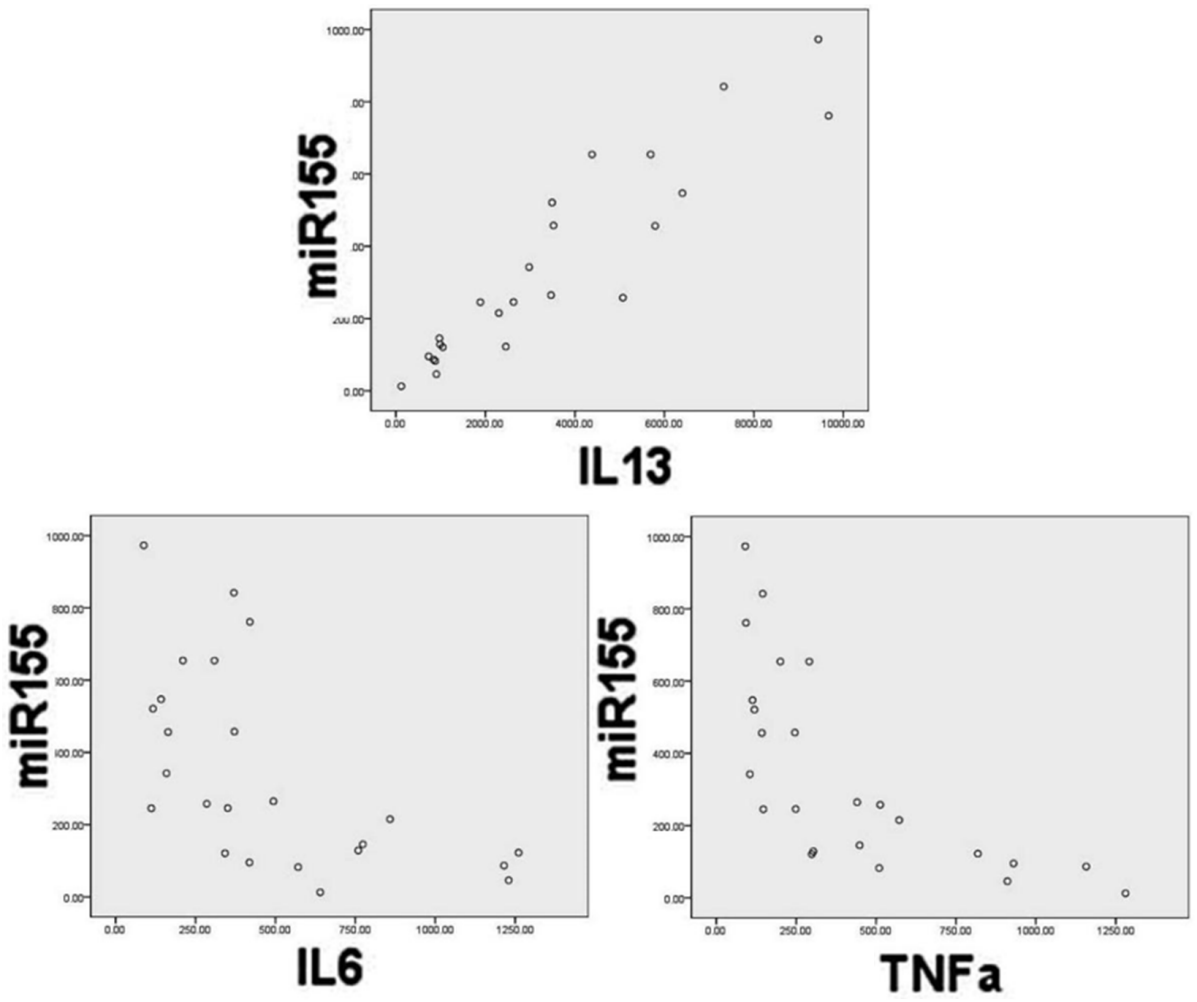
Correlation of miR-155 expression with IL-13, IL-6, and TNFα levels in serum Pearson correlation analysis showed that IL-13 was positively correlated with miRNA-155 (correlation coefficient=−0.597, *p*<0.001), while TNF-α was negatively correlated with miRNA-155 (correlation coefficient=−0.696, *p*=0.002).

Using ELISA, we measured a spectrum of inflammatory factors in the same group of NKTCL patients and subjected these data to correlation analysis with expression of miRNA-155. Expression of IL-6, IL-13, and TNF-α in sera and cell lines was correlated with the expression of miRNA-155 (P<0.05). Specifically, IL-13 was positively correlated, while IL-6 and TNF-α were negatively correlated, with miRNA-155 expression (Figure 3). These results indicate that miRNA-155 may regulate or influence inflammatory factors involved in NKTCL.

### miR-155 expression after infection with recombinant lentivirus

**Figure 4:**
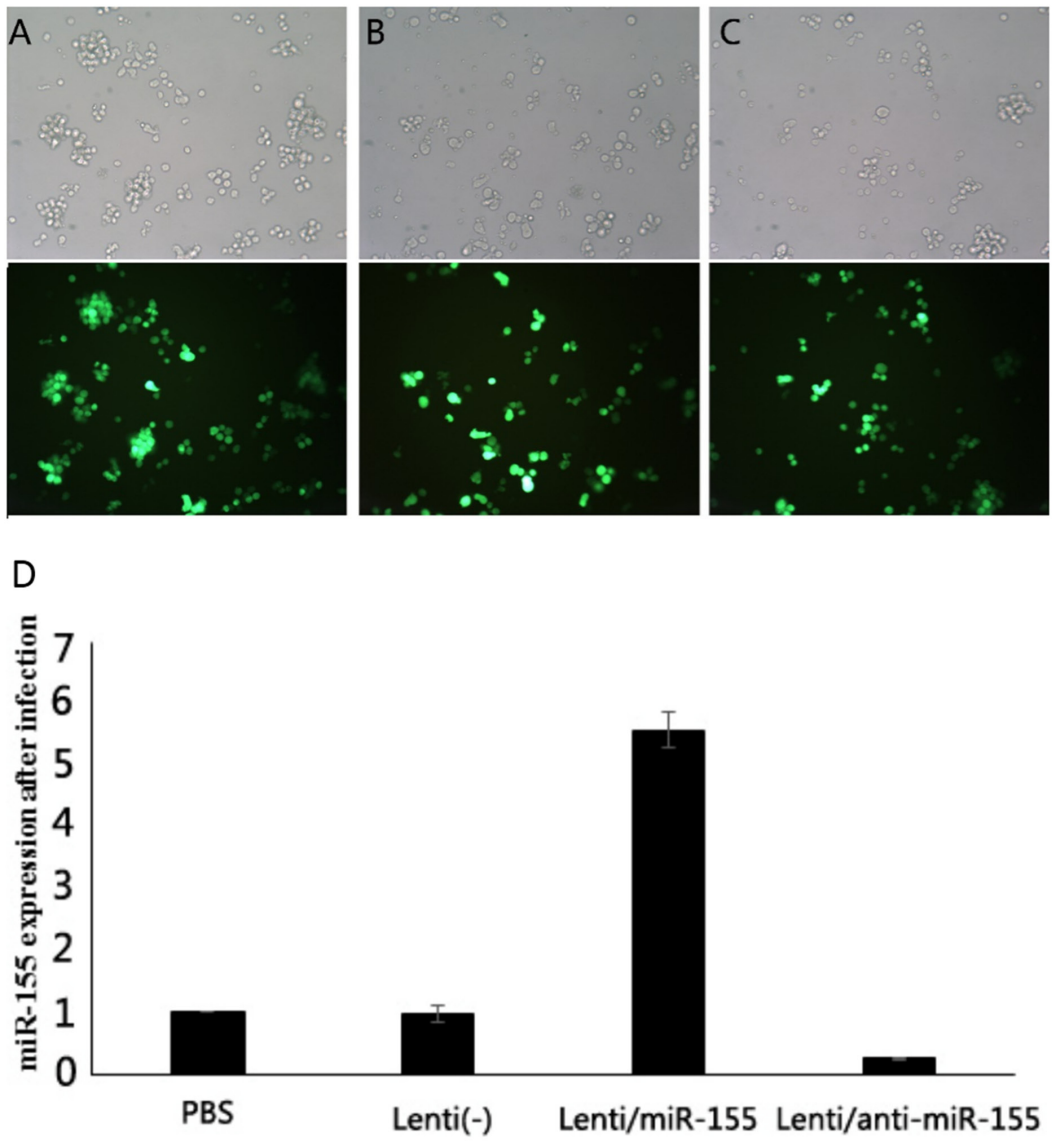
Fluorescence microscopy of GFP expression in infected human lymphoma cells SNK-6 cells were infected with Lenti (-), Lenti-miR-155, or Lenti-anti-miR-155. **A-C.** The infection rates of these three viruses were similar with approximately 80% infection efficiency (p> 0.05). **D.** miR-155 expression was higher in Lenti-miR-155 infected cells than these control cells, and down-regulated in Lenti-anti-miR-155 (p<0.05).

SNK-6 cells were infected with Lenti (-), Lenti-miR-155, and Lenti-anti-miR-155 at MOI 100. GFP expression verified that the infection status of these three viruses were similar with approximately 80% infection efficiency (p> 0.05). RT-PCR was used to evaluate miR-155 expression. Lenti(-) cells had a relative expression of 0.982±0.144, Lenti-miR-155 infected cells had a relative expression of 5.587±0.287, and Lenti-anti-miR-155 infected cells had expression of 0.253±0.019 compared to cells in PBS. Thus, miR-155 expression was higher in Lenti-miR-155 infected cells than in control cells (p<0.05), and down-regulated in Lenti-anti-miR-155 infected cells (Figure 4).

### Flow cytometric analysis of apoptosis

**Figure 5:**
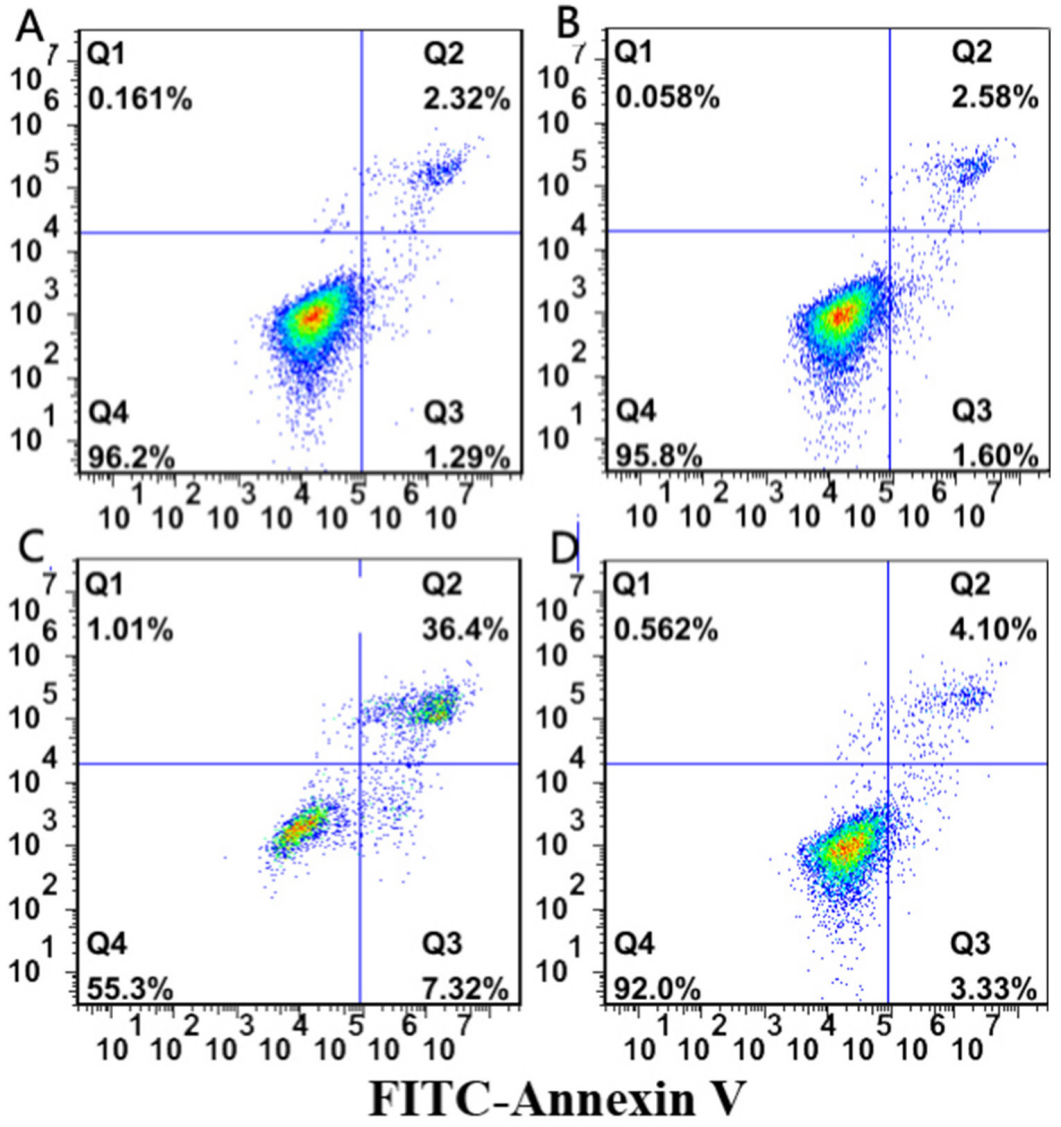
Flow cytometry assay results In order to explore the effects of miR-155 on apoptosis of NK/T lymphoma cells, flow cytometry was performed for four successive days in four groups. When miR-155 expression was downregulated by Lenti-anti-miR-155, the apoptosis rate of cells was higher than the Lenti-miR-155 and Lenti (-) groups (P<0.05).

In order to explore the effects of miR-155 on apoptosis of NKTCL cells, flow cytometry was performed for four successive days in four groups: PBS, Lenti(-), Lenti-anti-miR-155, Lenti-miR-155. The percentages of apoptosis were: 3.45±0.458, 3.15±0.568, 34.15±3.485, 7.45±1.226, respectively. Thus, when miR-155 expression was downregulated the apoptosis rate of Lenti-anti-miR-155 infected cells was higher than the Lenti-miR-155 and Lenti (-) groups (P<0.05; Figure 5).

### Cytotoxicity assay

**Figure 6:**
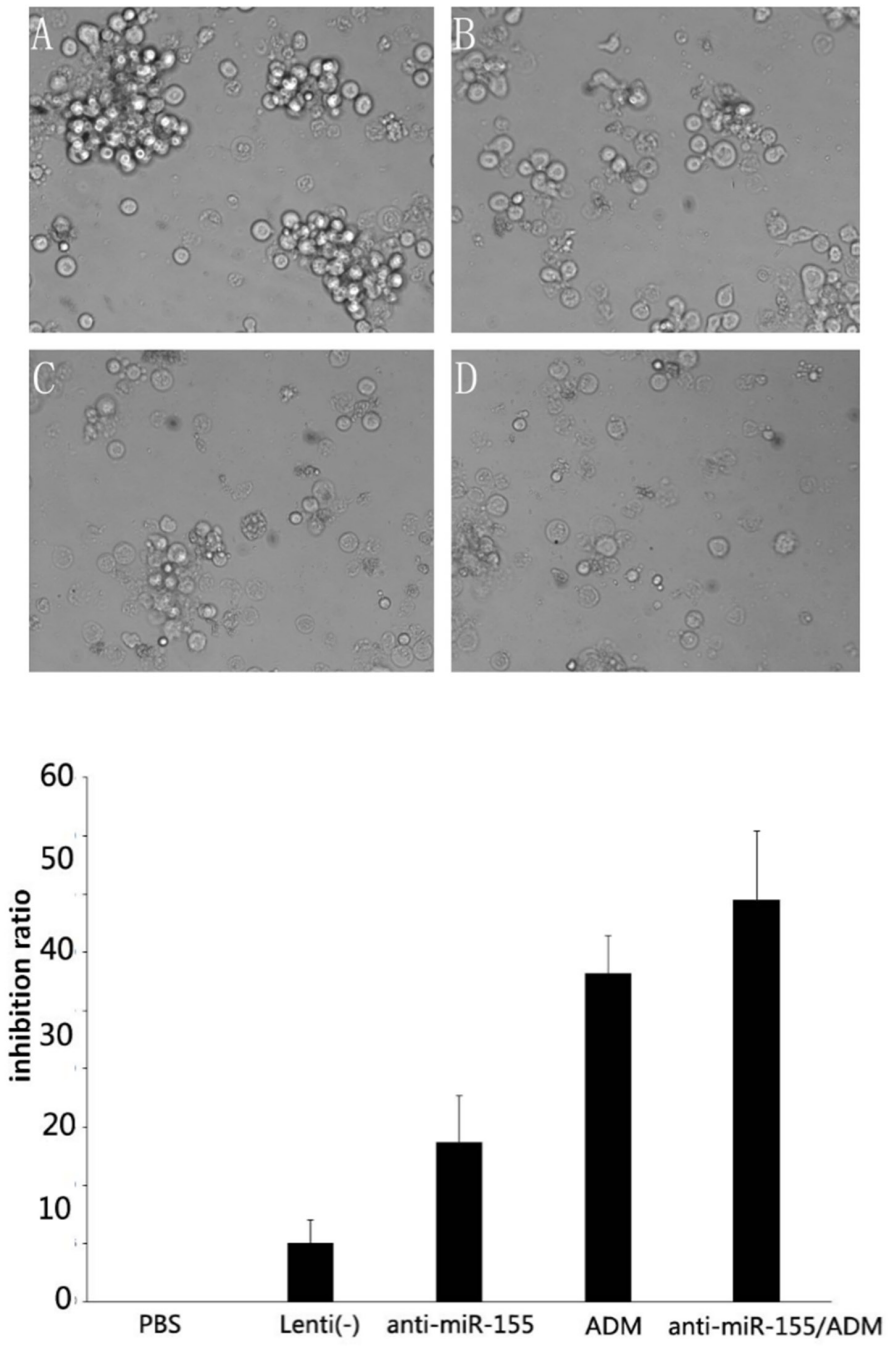
Cytotoxicity assay results SNK-6 cells were infected with recombinant lentivirus co-incubated with ADM at the concentration of 2.0 μg/ml (IC50=6.82μg/ml) for 48 h. The suppression rates in **A.** Lenti(-), **B.** Lenti-anti-miR-155, **C.** ADM, and **D.** ADM + Lenti-anti-miR-155 are shown, and suggest that the inhibition efficacy of SNK-6 cells was enhanced by combination treatment compared to ADM alone (P<0.05).

SNK-6 cells were infected with recombinant lentivirus co-incubated with ADM at a concentration of 2.0μg/ml (IC50=6.82μg/ml) for 48 h. The suppression rates in PBS, Lenti(-) (A), Lenti-anti-miR-155 (B), ADM (C), ADM + Lenti-anti-miR-155 (D) were 0, 5.07±1.99, 13.67±4.03, 28.21±3.23, 53.28±5.77, respectively. The effects of the two treatments were detected by MTT and FCA assay. In the MTT the inhibition efficacy of SNK-6 cells was enhanced by the combination treatment when compared with ADM treatment alone (P<0.05). Therefore, Lenti-anti-miR-155 had chemosensitizing activity in combination with ADM in SNK-6 cells (Figure 6).

### Evaluation of miR-155 target protein

**Figure 7:**
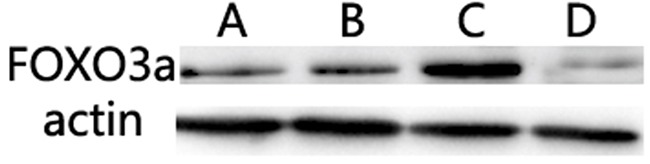
Evaluation of miR-155 target proteins The expression of FOXO3a in the Lenti-anti-miR-155 group was higher than in the other two groups, and lower in Lenti-miR-155 infected cells (P<0.05).

Expression of FOXO3a in the Lenti-anti-miR-155 group was higher than that in the other two groups, and lower in Lenti- miR-155 infected cells (P<0.05; Figure 7).

## DISCUSSION

The miRNA-155 mediates and regulates inflammation, controls the growth of immune cells, and is also closely associated with the differentiation and maturation of tumor cells [12]. A previous study showed that, along with protein kinase JNK and transcriptional regulators, such as NF-kappa B and AP-1, and the stimulation of inflammatory mediators, the expression of miRNA-155 in mature B and T lymphocytes rapidly increased. This increase suggested that miRNA-155 is important for the biological functions of lymphocytes [13]. miRNA-155 is one of the first miRNAs to be confirmed to promote cancer. It is highly expressed in most blood system malignant tumors, such as diffuse large B-cell lymphoma, adult T-cell leukemia, acute myeloid leukemia, chronic lymphocytic leukemia, Burkitt's lymphoma, and mantle cell lymphoma, as well as in some solid tumors, such as lung cancer and breast cancer [14-17]. Interestingly, miRNA-155 may play an important role in the development of NKTCL/leukemia, but not in B-cell lymphoma. When 10 NKTCL cell lines and tissue specimens from NKTCL 10 patients were tested, the expression of miRNA-155 was increased compared to control cells, and was related to the activation of EB virus infection and some transcription factors [5]. Furthermore, when miRNA-155 expression was reduced with antisense nucleic acids, its downregulation induced the increased SHIP expression and the downregulation of phosphorylated AKT. These findings indicated that miRNAs might play an important role in NKTCL by regulating signaling pathways.

In this study, we applied high-throughput sequencing technology to sequence the miRNAs of normal NK cells and two NKTCL cell lines; we then screened the differentially expressed miRNAs. We focused on analyzing the miRNAs that might be closely associated with the development of lymphoma or that were particularly abundant and differed markedly in their expression. To validate the high-throughput sequencing technology, we selected miRNA34a, miRNA34b, miRNA34c, miRNA-155, miRNA21, and miRNA221 in the cell lines for quantitative measurement by real-time PCR. The relative expression of miRNA-34a, miRNA-34b, miRNA-21, and miRNA-155 in SNK-6 cells and YTS cells were consistent with the results of miRNA sequencing. However, the relative expression of miRNA-155 in SNK-6 cells was higher than that in NK cells, even more than that in YTS cells, which indicated that miRNA-155 may play an important role in SNK-6 cells. In support of this, our previous study confirmed that miRNA-155 affects the biological activity of NKTCL.

Free miRNA in serum is generally considered to originate from apoptotic or necrotic cells, including tumor cells, but active release by growing cells is another source of miRNAs. Relative miRNA levels can be detected in serum or body fluid from the cells in circulation, especially the lysis of cells in the lymphatic hematopoietic system [18-20]. miRNA-21 was the first miRNA marker in serum to be detected. Its levels are high in patients with diffuse large B-cell lymphoma, and correlate with recurrence-free survival [14]. Other miRNAs in circulation can also be detected in relation to solid tumors, such as bladder cancer [21], colon cancer [22], and breast cancer [23], as well as in blood lymphatic system tumors, such as leukemia [24] and lymphoma [25]. These miRNAs are also correlated with clinical factors, such as etiology, therapeutic effects, prognosis, and risk. Therefore, they might be useful clinically as oncogenic molecular markers and targets.

Persistent inflammation can promote cancer, and the continued upregulation of miRNA-155 not only leads to a sustained inflammatory response, but also promotes tumor development [26]. Almost all patients with nasal NKTCL have suffered chronic rhinitis or sinusitis for years; the rate of EB virus infection in such cases has been reported to be 80%–100%. EB virus infection not only promotes and aggravates chronic rhinitis and sinusitis, but also promotes the transformation of inflammatory lesions to malignant ones, which can eventually develop into nasal NKTCL. We confirmed that miR-155 plays an important role in regulating immune responses to and the development of viral infections, indicating that this miRNA can promote the development of tumors by regulating the expression of inflammatory factors and changing the microenvironment. Accordingly, miRNA-155 may act as a bridge between inflammatory and malignant tumors [27, 28]. In this study, we found that many kinds of inflammatory factors, such as IL-6, IL-13, and TNF, were abnormally expressed in NKTCL cells and correlated with the expression of miRNA-155. Our previous research confirmed that FOXO3a is a target of miRNA-155. In this study using bioinformatic technology, we found that IL-6R, SOS-1, NF-IL-6, c-Fos, MAP3K10, NIK, SP1, MEF2, and other inflammatory factors and tumor regulatory molecules are also potential target genes of miRNA-155, but no definitive results have been reported yet. These findings further indicate that miRNA-155 may play an important role in inflammatory and malignant tumors.

Chemotherapy in NKTCL is often ineffective, especially in those cases not sensitive to CHOP treatment [29]. This is probably because of the high proliferative activity of tumor cells and low sensitivity to chemotherapeutics. miRNAs can improve the sensitivity of tumor cells to chemotherapeutics or reverse the resistance to drugs [30]. We used lentiviral vectors to infect SNK-6 cells [31] in order to modulate the expression of miR-155. Inhibition of miR-155 increased cell apoptosis, which suggested that the increased expression of miR-155 in NKTCL might inhibit cell apoptosis. Doxorubicin is a traditional drug to lymphoma, but NKTCL isn’t sensitive to doxorubicin [32]. We showed that the inhibition efficiency of SNK-6 cells with ADM alone at the concentration of 2.0 μg/ml was lower than 30%. However, combination treatment with the Lenti-anti-miR-155 group increased the inhibition efficacy, suggesting that inhibition of miR-155 expression could improve the sensitivity of NKTCL to doxorubicin, even reverse the drug resistance.

Overall, we show that miRNA-155 is highly expressed in NKTCL cells, and as an oncogene. All of these findings suggest that miRNA-155 is a molecular target with tremendous potential in NKTCL research. In the future, we plan to conduct an in-depth study on the effects and molecular mechanisms of the malignant biological behaviors of NKTCL cells with or without induction by inflammatory factors. We hope to identify effective targets and molecular markers for the clinical diagnosis and treatment of NKTCL.

## MATERIALS AND METHODS

### Cell lines

The human NKTCL cell lines SNK-6 and YTS were kindly provided by Professor Norio Shimizu and Professor Yu Zhang of Chiba University (Chiba, Japan), and Dr. Daniel Billadeau from the Mayo Clinic (Rochester, MN, USA), respectively. YTS cells were grown in RPMI-1640 with 10% fetal bovine serum and 0.1 mM nonessential amino acids. SNK-6 cells were grown in RPMI-1640 with 10% human serum and 700 U/ml IL-2. Normal NK cells were supplied by the Biotherapy Center of the First Affiliated Hospital of Zhengzhou University.

### Clinical data

Between September 2011 and January 2013, 20 patients were diagnosed with NKTCL at the Department of Oncology, First Affiliated Hospital of Zhengzhou University; these patients were included in this study along with 10 healthy volunteers as controls. The lymphoma stage was assessed using the Ann Arbor system and the modified Ann Arbor staging system for nasal NKTCL [33] via computed tomography (CT) scans or positron emission tomography/CT. Performance status was evaluated based on the Eastern Cooperative Oncology Group scale [34]. International Prognostic Index scores were used to determine the risk classification [35]. Treatment responses including complete remission (CR), partial remission (PR), stable disease (SD), and progressive disease (PD) were evaluated in accordance with the response criteria of Cheson [36]. Peripheral blood serum was separated before each session of chemotherapy or initial treatment. All patients provided informed consent. Patients with incomplete clinical or pathological data were excluded from this study. In the healthy controls, chronic infection and autoimmune disease were ruled out. All pathological specimens were validated by three experienced pathologists in accordance with WHO classifications. Follow-up was conducted every 2–3 weeks.

### RNA processing

Total RNA was extracted from cell lines and plasma using TRIzol reagent (Invitrogen, Carlsbad, CA, USA), in accordance with the instructions provided with the miRNeasy Serum/Plasma Kit (217184, Qiagen) and the RNeasy Mini Kit (Qiagen), after which it was homogenized and pooled for library construction and Solexa sequencing. The quantity and integrity of total RNA were assessed using an Agilent RNA 6000 Nano Kit (5067-1511, Agilent Technologies, USA). Total RNA was stored at −80°C until further use.

### Small RNA sequencing

The sequencing platform for Solexa high-throughput sequencing was used for miRNA expression profiling at Beijing Genomics Institute (BGI), Shenzhen, China. We used this next-generation deep sequencing technology on the NKTCL cell lines SNK-6 and YTS, as well as NK cells as a normal control. The small RNA sequencing procedure used ‘sequencing by synthesis’ to identify the small molecule RNAs of samples and discover new small molecule RNAs, as well as to establish the difference in the expression profile of small RNAs between samples. This process involved the following steps: small RNA being isolated from total RNA, 5′ header purification, 3′ header purification, reverse-transcription polymerase chain reaction (RT-PCR) amplification, purification of small RNA libraries, library detection, DNA amplification clustered in Cluster Station, and sequencing on an Illumina Genome Analyzer.

### Bioinformatic analysis

The sequence data were analyzed after treatment to remove joints, low-quality data, and pollution, so as to obtain a credible target sequence for alternative analysis, and to calculate the sequence length distribution and public sequence among the samples. Once all of the small RNA fragments had been annotated, the following available biological information was collated: the results of miRNA difference analysis, cluster analysis, miRNA target gene prediction, and other advanced biological analyses. Information was obtained from the following sources: miRNA database: miRNAbase (http://www.miRNAbase.org); gene annotation database: Gene Ontology (GO) database (http://www.geneontojogy.org); miRNA comparison: tag2miRNA (BGI); repeat sequence alignment: tag2repeat (BGI); homology comparison software: BLAST (http://blast.ncbi.nlm.nih.gov/Blast.cgi); small RNA taxon notes: tag2annotation (BGI); miRNA target gene prediction software tool: Targetscan online miRNA prediction; GO term analysis software: OBO-editor; and online primer design software: primer3 (http://frodo.wi.mit.edu/primer3/).

### Reverse transcription and quantitative real-time PCR

Initially, 0.2–0.5 μg of total RNA was reverse-transcribed to cDNA using a target-specific stem-loop primer (Supplementary Table S1). Quantitative (q) PCR was performed on a sequence detection system (Prism 7500; Applied Biosystems Inc., Foster City, CA, USA). In brief, cDNA in water was added to 5 μL of 2×SYBR Green Master Mix (DRR041A; Takara, Tokyo, Japan) and 400 nmol/L gene-specific primers, and then water was added to 10 μL. The reactions were amplified at 95°C for 10 min, followed by 40 cycles at 95°C for 15 s and 60°C for 60 s. U6 small nuclear RNA (U6) served as an endogenous control. At the end of the qPCR, the thermal denaturation protocol was run to determine the number of products that were present in the reaction. The relative amount of miRNA to U6 was calculated using the Ct cycle method. The relative amount of each miRNA to U6 was described using the following formula: ΔΔCt =[Ct_(target gene)_ − Ct_(U6)_]_target cell_ − [Ct_(target gene)_ − Ct(_U6_)]_reference cell_ [32,37].

### Enzyme-linked immunosorbent assay (ELISA)

A 96-well plate was incubated with 100 μl of carbonate coating buffer, in which antigen (50 mM, 10-20 μg/ml) was dissolved, overnight at 4°C. The next morning, the wells were washed (0.05% Tween20 in phosphate-buffered saline) and blocked on 150 μl of 1% bovine serum albumin for 1 h at 37°C. After washing the wells again, the samples and standards were added to the 96-well plate and incubated for 2 h at room temperature. Detection antibody was added and incubated for 2 h at 25°C. Streptavidin–horseradish peroxidase solution was added to each well for 20 min and substrate solution was used to visualize the reaction. After 20 min in the substrate solution, stop solution was added to the reaction and the protein concentration was determined using a standard curve at 450 nm by nanospectroscopy.

### miR-155 expression after infection with recombinant lentivirus

SNK-6 cells were infected with lentivirus at multiplicity of infection (MOI) of 100, and Total RNA was extracted 96 h after infection. Real-time PCR was performed to evaluate the regulation of miR-155 expression by recombinant lentivirus.

### Flow cytometry assay

We seeded 1 × 10^6^ cells in 6 cm dishes and infected with Lenti (-), Lenti-miR-155 and Lenti-anti-miR-155 for 48 h, then cells were collected and apoptosis analyzed by FACScan.

### Cytotoxicity assay of Lenti-anti-miR-155 combined with Doxorubicin (ADM)

SNK-6 cells were infected with recombinant lentivirus in 6-well plates for 96 h and then reseeded in 96-well plates at an optimal density (1 × 10^4^ cells per well), the cells were co-incubated with ADM at the concentration of 2.0 μg/ml (IC50=6.82μg/ml) for 48 h. Cytotoxicity effects of Lenti-anti-miR-155 combined with ADM were detected by MTT.

### Evaluation of miR-155 target protein

Software predictions and our previous study showed that FOXO3a was a potential target of miRNA-155 [31]. To determine its target, proteins were extracted from SNK-6 cells infected by recombinant lentiviruses. Western blotting was used to detect the protein expression of FOXO3a.

### Statistical analysis

Differences of miRNA expression were analyzed using normalized fold change and P-value. Fold change (log_2_NK/SNK-6 or YTS) of >1.0 or <−1.0 and p-value <0.01 were considered significant. Associations between miRNA-155 expression and clinical parameters were assessed by Fisher's exact or X^2^ test. Data are presented as mean ± standard deviation. SPSS 17.0 statistical software package was used for statistical analysis of independent sample data using *t*-test. A p-value of <0.05 was considered to be statistically significant, and all p-values correspond to two-sided significance tests.

## SUPPLEMENTARY MATERIALS FIGURES AND TABLES




