# Supplementary material for: MicroRNA-155 is a potential molecular marker of natural killer/T-cell lymphoma
Source: Oncotarget. 2016 Jul 22;7(33):53808–19. doi: 10.18632/oncotarget.10780 (PMC5288223; doi:10.18632/oncotarget.10780)
Supplement: Supplementary file 1 [file oncotarget-07-53808-s001.pdf]

MicroRNA-155 is a potential molecular marker of natural killer/T-cell lymphoma

SUPPLEMENTARY FIGURE AND TABLES

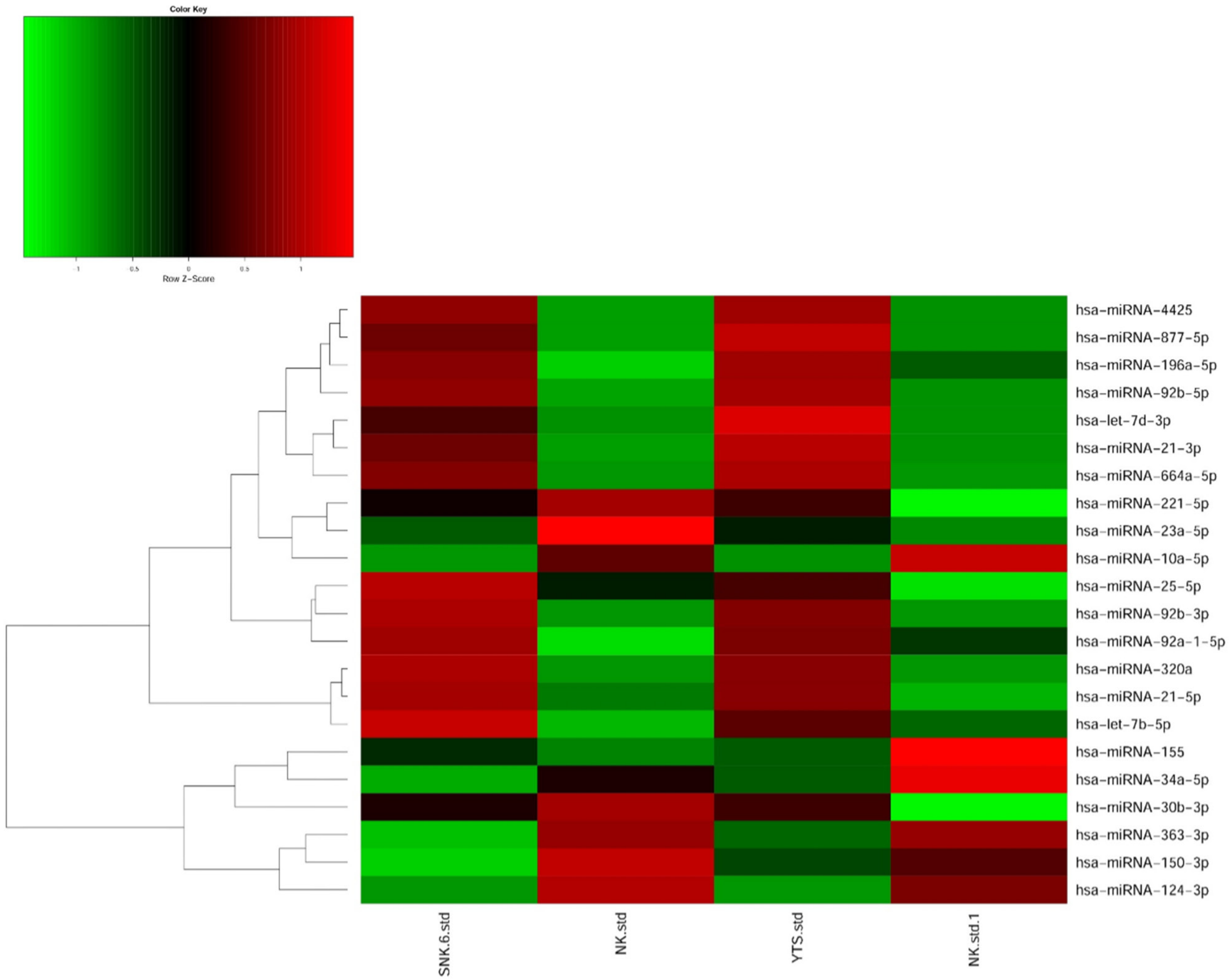

Supplementary Figure S1: Heatmap of differential expression of small RNAs in NK, SNK-6, and YTS cells

Supplementary Table S1: Summary of miRNA primers used in real-time RT-PCR

name	sequence 5′ -3′
miRNA-155-RT	GTCGTATCCAGTGCAGGGTCCGAGGTATTCGCACTGGATACGACACCCCT
miRNA-155-qPCR-f	GCGCCGTTAATGCTAATCGTGAT
miRNA-155-qPCR-r	GTGCAGGGTCCGAGGT
miRNA221-RT	GTCGTATCCAGTGCAGGGTCCGAGGTATTCGCACTGGATACGACGAAACC
miRNA221-qPCR-f	GTCTCGAGCTACATTGTCTGCTG
miRNA221-qPCR-r	GTGCAGGGTCCGAGGT
miRNA21-RT	GTCGTATCCAGTGCAGGGTCCGAGGTATTCGCACTGGATACGACTCAACA
miRNA21-qPCR-f	CCCGCGTAGCTTATCAGACTGA
miRNA21-qPCR-r	GTGCAGGGTCCGAGGT
miRNA34a-RT	GTCGTATCCAGTGCAGGGTCCGAGGTATTCGCACTGGATACGACACAACC
miRNA34a-qPCR-f	GCTGCCTGGCAGTGTCTTAG
miRNA34a-qPCR-r	GTGCAGGGTCCGAGGT
miRNA34b-RT	GTCGTATCCAGTGCAGGGTCCGAGGTATTCGCACTGGATACGACCAATCA
miRNA34b-qPCR-f	CCGGGCAGGCAGTGTCAT
miRNA34b-qPCR-r	GTGCAGGGTCCGAGGT
miRNA34c-RT	GTCGTATCCAGTGCAGGGTCCGAGGTATTCGCACTGGATACGACGCAATC
miRNA34c-qPCR-f	GCCCCGAGGCAGTGTAGT
miRNA34c-qPCR-r	GTGCAGGGTCCGAGGT
RNT6-RT	GTCGTATCCAGTGCAGGGTCCGAGGTATTCGCACTGGATACGACACAAAATATGGAACGCTT
RNT6-qPCR-f	GTGCTCGCTTCGGCAGCACA
RNT6-qPCR-r	AACGCTTCACGAATTTGCGT

Supplementary Table S2: Expression of candidate miRNAs in SNK-6 cells and NK cells

miRNA-name	SNK-6-std	NK-std	fold-change (log2 SNK-6/NK)	*p*-value
hsa-miRNA-1246	2997.7324	8.0255	8.54506	0
hsa-miRNA-92b-5p	522.2202	19.6624	4.73115	0
hsa-miRNA-92a-1-5p	2855.4799	139.3756	4.35668	0
hsa-miRNA-340-5p	148.7712	7.8917	4.23662	1.78E-246
hsa-miRNA-92b-3p	1293.3099	80.5222	4.00554	0
hsa-miRNA-424-3p	170.2105	12.0382	3.82163	4.24E-261
hsa-miRNA-196a-5p	286.6779	24.4777	3.54989	0
hsa-miRNA-4425	470.7949	57.7834	3.02637	0
hsa-miRNA-664a-5p	64.4627	8.1592	2.98197	3.65E-80
hsa-miRNA-21-3p	137.4721	17.5223	2.97187	2.13E-168
hsa-miRNA-21-3p	137.4721	17.5223	2.97187	2.13E-168
hsa-miRNA-503-5p	182.6684	24.6114	2.89183	5.60E-217
hsa-miRNA-532-3p	42.5888	5.8853	2.85529	3.09E-51
hsa-miRNA-320a	34998.8947	5879.8572	2.57346	0
hsa-miRNA-92a-3p	3525.6021	631.7379	2.48047	0
hsa-miRNA-21-5p	29169.8741	6155.6657	2.24449	0
hsa-miRNA-877-5p	304.3508	65.8088	2.20938	1.91E-264
hsa-miRNA-25-5p	2223.0212	519.9164	2.09617	0
hsa-let-7b-5p	19029.386	4499.0759	2.08053	0
hsa-miRNA-17-3p	269.5844	64.4712	2.06401	4.58E-215
hsa-miRNA-320c	70.6917	17.121	2.04577	4.27E-57
hsa-miRNA-128	1283.3145	319.146	2.00759	0
hsa-miRNA-155	3.1869	0.9363	1.76711	0.002624
hsa-miRNA-32-5p	8.257	2.6752	1.62597	3.89E-06
hsa-let-7d-3p	99.6637	32.9044	1.59879	3.13E-57
hsa-miRNA-30b-3p	3.9112	8.0255	-1.03698	0.00143377
hsa-miRNA-148a-3p	2.8972	6.8216	-1.23545	0.00073543
hsa-miRNA-221-5p	50.4113	140.4456	-1.47819	1.81E-70
hsa-miRNA-15a-5p	21.5841	76.3757	-1.82314	3.78E-52
hsa-miRNA-625-3p	15.9346	75.4394	-2.24316	6.19E-67
hsa-miRNA-10a-5p	14.3411	71.8279	-2.32439	1.48E-66
hsa-miRNA-23a-5p	23.4673	198.3628	-3.07942	3.93E-245
hsa-miRNA-150-3p	0.01	79.8534	-12.96314	2.71E-170
hsa-miRNA-363-3p	0.01	88.6814	-13.11442	4.84E-189
hsa-miRNA-124-3p	0.01	419.4643	-15.35626	0
hsa-miRNA-34a-5p	0.1449	6.8216	-5.55698	8.80E-14

Supplementary Table S3: Candidate miRNA expression in YTS cells and NK cells

See Supplementary File 1
