# Supplementary material for: MicroRNA-155 is a potential molecular marker of natural killer/T-cell lymphoma
Source: Oncotarget. 2016 Jul 22;7(33):53808–19. doi: 10.18632/oncotarget.10780 (PMC5288223; doi:10.18632/oncotarget.10780)
Supplement: Supplementary file 2 [file oncotarget-07-53808-s002.docx]

**Supplementary table 3. Candidate miRNA expression in YTS cells and NK cells**

| miRNA-name | YTS-std | NK-std | fold-change (log2 YTS/NK) | *p*-value |
| --- | --- | --- | --- | --- |
| hsa-miRNA-125b-5p | 936.1489 | 0.01 | 16.51444518 | 0 |
| hsa-miRNA-383 | 355.1644 | 0.01 | 15.11619808 | 0 |
| hsa-miRNA-204-3p | 173.4441 | 0.01 | 14.08218224 | 0 |
| hsa-miRNA-99a-5p | 97.8948 | 0.01 | 13.25701611 | 1.88E-228 |
| hsa-miRNA-3689a-5p | 59.9429 | 0.01 | 12.54937342 | 3.71E-140 |
| hsa-miRNA-3689b-5p | 59.9429 | 0.01 | 12.54937342 | 3.71E-140 |
| hsa-miRNA-3689e | 59.9429 | 0.01 | 12.54937342 | 3.71E-140 |
| hsa-miRNA-100-5p | 219.7905 | 0.1338 | 10.68183526 | 0 |
| hsa-miRNA-125a-5p | 1.7735 | 0.01 | 7.47045552 | 7.94E-05 |
| hsa-let-7c | 9047.4691 | 186.8596 | 5.59748769 | 0 |
| hsa-miRNA-22-3p | 610.5422 | 19.2611 | 4.98632901 | 0 |
| hsa-miRNA-196a-5p | 659.7261 | 24.4777 | 4.75232727 | 0 |
| hsa-miRNA-449c-5p | 46.5828 | 2.4076 | 4.27412973 | 7.53E-84 |
| hsa-miRNA-30d-5p | 2080.147 | 145.2609 | 3.83996716 | 0 |
| hsa-miRNA-92b-5p | 251.4762 | 19.6624 | 3.67691053 | 0 |
| hsa-miRNA-30b-3p | 91.6286 | 8.0255 | 3.51313479 | 4.41E-141 |
| hsa-miRNA-92b-3p | 864.0283 | 80.5222 | 3.42362007 | 0 |
| hsa-miRNA-505-5p | 58.5241 | 5.6178 | 3.38095367 | 8.76E-88 |
| hsa-miRNA-664a-5p | 69.8742 | 8.1592 | 3.09826025 | 1.65E-96 |
| hsa-miRNA-7-5p | 144.8323 | 17.5223 | 3.04711932 | 5.03E-195 |
| hsa-miRNA-877-5p | 530.6184 | 65.8088 | 3.01132228 | 0 |
| hsa-miRNA-92a-1-5p | 1013.4717 | 139.3756 | 2.86225588 | 0 |
| hsa-miRNA-10a-5p | 496.2133 | 71.8279 | 2.78834416 | 0 |
| hsa-let-7d-3p | 221.9186 | 32.9044 | 2.75367817 | 1.25E-268 |
| hsa-let-7d-5p | 19357.6396 | 2879.537 | 2.74899428 | 0 |
| hsa-miRNA-532-5p | 94.2297 | 14.4458 | 2.70553175 | 5.51E-113 |
| hsa-let-7b-5p | 25803.0923 | 4499.0759 | 2.51984336 | 0 |
| hsa-miRNA-4425 | 327.4984 | 57.7834 | 2.50276086 | 0 |
| hsa-miRNA-760 | 97.4219 | 19.1274 | 2.34860534 | 1.18E-99 |
| hsa-miRNA-766-5p | 168.4784 | 33.5732 | 2.32718168 | 3.58E-169 |
| hsa-miRNA-320a | 28963.0383 | 5879.8572 | 2.30035993 | 0 |
| hsa-miRNA-1301 | 68.219 | 14.1783 | 2.26648905 | 2.02E-67 |
| hsa-let-7e-5p | 395.7174 | 84.1336 | 2.23371652 | 0 |
| hsa-miRNA-1307-3p | 1209.3796 | 271.2607 | 2.1565152 | 0 |
| hsa-miRNA-378c | 101.6782 | 23.9426 | 2.08635866 | 4.92E-90 |
| hsa-miRNA-25-5p | 2200.0327 | 519.9164 | 2.0811734 | 0 |
| hsa-let-7a-5p | 156175.9582 | 38314.507 | 2.0272097 | 0 |
| hsa-miRNA-21-5p | 12721.8358 | 6155.6657 | 1.04732008 | 0 |
| hsa-miRNA-21-3p | 35.3509 | 17.522 | 1.01255479 | 3.99E-12 |
| hsa-miRNA-155 | 4.6984 | 0.9363 | 2.32712678 | 0 |
| hsa-miRNA-24-3p | 87.1359 | 402.6108 | -2.20804674 | 0 |
| hsa-miRNA-23a-3p | 79.9238 | 626.3876 | -2.97035857 | 0 |
| hsa-miRNA-27a-3p | 21.5179 | 171.8787 | -2.99778158 | 1.23E-235 |
| hsa-miRNA-221-5p | 15.0153 | 140.4456 | -3.22550622 | 3.16E-207 |
| hsa-miRNA-221-3p | 81.4608 | 1104.1703 | -3.76071291 | 0 |
| hsa-miRNA-222-3p | 35.1145 | 690.0563 | -4.29657527 | 0 |
| hsa-miRNA-34a-5p | 0.2365 | 6.8216 | -4.85019807 | 6.78E-15 |
| hsa-miRNA-146a-5p | 2.2464 | 81.726 | -5.18510838 | 8.90E-171 |
| hsa-miRNA-23a-5p | 1.6552 | 198.3628 | -6.90499213 | 0 |
| hsa-miRNA-124-3p | 0.7094 | 419.4643 | -9.20773298 | 0 |
| hsa-miRNA-363-3p | 0.1182 | 88.6814 | -9.5512577 | 4.22E-216 |
| hsa-miRNA-150-3p | 0.01 | 79.8534 | -12.96313812 | 5.86E-197 |
